# Personalized Virus Load Curves for Acute Viral Infections

**DOI:** 10.3390/v13091815

**Published:** 2021-09-13

**Authors:** Carlos Contreras, Jay M. Newby, Thomas Hillen

**Affiliations:** 1 Department of Mathematical and Statistical Sciences, University of Alberta, Edmonton, AB T6G 2R3, Canada; carlos.contreras@ualberta.ca (C.C.); jnewby@ualberta.ca (J.M.N.); 2 Collaborative Mathematical Biology Group, University of Alberta, Edmonton, AB T6G 2R3, Canada

**Keywords:** viral load, patient specific, mathematical modeling, SARS-CoV-2

## Abstract

We introduce an explicit function that describes virus-load curves on a patient-specific level. This function is based on simple and intuitive model parameters. It allows virus load analysis of acute viral infections without solving a full virus load dynamic model. We validate our model on data from mice influenza A, human rhinovirus data, human influenza A data, and monkey and human SARS-CoV-2 data. We find wide distributions for the model parameters, reflecting large variability in the disease outcomes between individuals. Further, we compare the virus load function to an established *target model* of virus dynamics, and we provide a new way to estimate the exponential growth rates of the corresponding infection phases. The virus load function, the target model, and the exponential approximations show excellent fits for the data considered. Our virus-load function offers a new way to analyze patient-specific virus load data, and it can be used as input for higher level models for the physiological effects of a virus infection, for models of tissue damage, and to estimate patient risks.

## 1. Introduction

The ongoing global SARS-CoV-2 pandemic has stimulated new research on viral infection and transmission. COVID-19, the disease caused by SARS-CoV-2, has found an abundantly susceptible population with no previous immunities. The disease has infected more than 150,000,000 people world wide, with about 3,000,000 deaths (as of May 2021). While progressing mildly in most cases, severe cases show respiratory symptoms followed by complications in other tissues, such as cardiac tissue, blood vessels, kidney, digestive system and nervous system [1,2,3]. A good understanding of the viral progression inside a patient is of vital interest for the design of treatment and/or vaccination strategies.

For example, in a recent study of over 600 SARS-CoV-2 patients in France [4], a correlation was found between the maximal viral load of a patient and the severity of the disease. In SARS-CoV-2 infection, the maximal viral load arises early in the disease, typically before day four ([5,6]), and often patients are not even tested at that point in time; hence, the maximal viral load might be unknown. Mathematical models can help to estimate the viral load in a given patient (or animal). The standard mathematical model for virus load functions of acute viral infections is the *Baccam model* [7], which is also referred to as the *target model* [8]. It is a system of differential equations for viral load, target cells, and various levels of infected cells. We will discuss the target model and its extensions later in Section 2.5. Here, our approach is different. Based on preliminary work in [9], we propose an explicit function that can describe patient specific viral load curves based on available data. We do not attempt to criticize the target model framework; rather, we offer an alternative way to analyze the available data.

**Figure 1:**
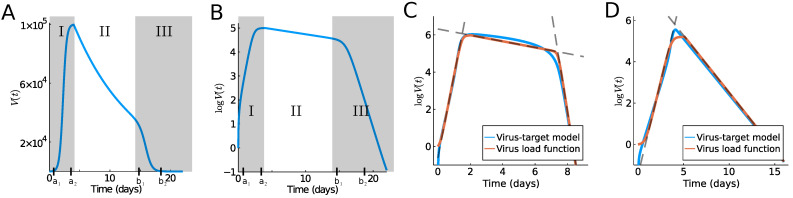
Typical virus load curves. The virus load (“titer”) is usually reported as a dilution value, TCID50, that is needed to infect 50% of a given cell culture in (**A**) absolute scale and (**B**) logarithmic scale. Shadow areas indicate the three phases into which we divide the virus load progression. Phase I describes the initial increase until a maximum is reached, Phase II denotes intermediate decay and Phase III, virus clearance. Images (**C**,**D**) show a comparison of the various models considered in this paper, where (**C**) shows a tri-phasic response and (**D**), a bi-phasic response. The red line shows the virus load function (1), the blue line, the corresponding solution of the target model ODE (9). The dashed lines indicate the linear approximations of the various phases, where phase I and III are computed by (8). Parameter values are given in Table 1 and Table 2.

While our research is certainly motivated by the current COVID-19 pandemic, similar dynamics are known for other acute viral infections, such as Influenza A [8] or MERS and SARS [2]. The progression of the viral load has a very typical time course, which can be classified into several temporal phases. Typically, an initial fast exponential increase leads to a virus load maximum (Phase I), which is followed by a slow exponential decrease (Phase II), followed by a fast exponential decrease, leading to clearance (Phase III) (see Figure 1). Determining the duration and speed of these phases is important in the understanding of the disease progression in a given patient. Here, we propose a simple model for the virus load that provides such biologically meaningful information. The model is based on intuitive, model parameters, such as the time of viral infection, the time to reach the maximum, and the time point of fast viral clearance toward the end of the infection. The result is an explicit virus load function that can be used in higher level models, which focus on the effect of a virus on the immune system, anti-viral therapies, assessments of tissues and organs damage, as well as person-to-person infectivities.

Virus load curves, as reported in [2,10,11], have a very typical infection progression (see Figure 1A,B). In A. Smith [10] the virus infection has been classified into five phases, which we will combine into three phases for our purpose. In Smith’s classification, in the first phase (Phase Ia) the virus quickly infects cells without being detectable. This phase is followed by exponential growth (Phase Ib) until growth shows signs of saturation and a maximum is reached (Phase Ic). A period of slow exponential decline ensues, which we call Phase II. And finally, we often observe a fast decline that leads to virus clearance (Phase III). Depending on the virus and the response of the infected individual, these phases can be shorter or longer. The last Phase III is sometimes not seen in patient data, and the virus is cleared before the third phase starts. It is useful to distinguish between a tri-phasic behavior as in Figure 1C versus a bi-phasic behavior as in Figure 1D.

To develop our virus load function below (see (1)), we consider Phase I of the sigmoid increase between time points $a_{1}$ and $a_{2}$ (see Figure 1A,B). Phase I includes the three initial phases (Phases Ia, Ib, Ic) of Smith [10] mentioned above. At time $a_{2}$, a slow decline of the virus is observed as the immune response kicks in (Phase II between $a_{2}$ and $b_{1}$ in Figure 1), and finally (Phase III) shows a rather sharp decline once the virus is controlled (between $b_{1}$ and $b_{2}$). Based on [9], we write the virus load curve as a product of three functions, representing the three main phases:

(1) $$V\left(t\right)=v_{1}\left(t\right)\;v_{2}\left(t\right)\;v_{3}\left(t\right),$$

where $v_{1}$ describes the initial growth phase between $a_{1}$ and $a_{2}$, $v_{2}$, the intermediate slow decay phase between $a_{2}$ and $b_{1}$, and $v_{3}$ the final decay phase between $b_{1}$ and $b_{2}$. These are given as sigmoid and exponential functions, respectively, as follows:

(2) $$\begin{array}{cc} v_{1}\left(t\right) & =1+\frac{V_{\max}-1}{2}\left[\tanh\left(\frac{6}{a_{2}-a_{1}}\left(t-\frac{a_{1}+a_{2}}{2}\right)\right)-\tanh\left(-3\;\frac{a_{2}+a_{1}}{a_{2}-a_{1}}\right)\right], \end{array}$$

(3) $$\begin{array}{cc} v_{2}\left(t\right) & =\left\{\begin{array}{cc} 1 & t<a_{2}\\ e^{-\alpha (t-a_{2})} & t\geq a_{2} \end{array}\right., \end{array}$$

(4) $$\begin{array}{cc} v_{3}\left(t\right) & =1-\frac{1-V_{\min}}{2}\left[\tanh\left(\frac{6}{b_{2}-b_{1}}\left(t-\frac{b_{1}+b_{2}}{2}\right)\right)-\tanh\left(-3\;\frac{b_{2}+b_{1}}{b_{2}-b_{1}}\right)\right]. \end{array}$$

The specific form of sigmoid curves for $v_{1}$ and $v_{3}$ was developed previously by Olobatuyi in [12] in a cancer model, and more details are given in Section 2. It allows us to define these functions based on intuitive transition threshold values. The value $a_{1}$ describes the onset of growth, and $a_{2}$ a value when saturation is reached; similarly, $b_{1}$ denotes the time where decay switches from slow to fast, and $b_{2}$ is the time when the virus is effectively eliminated. The parameter α describes the intermediate exponential decay rate. In Table 1, we list the values used in Figure 1A,B and their meaning. This virus load function can describe the tri-phasic or bi-phasic response, as shown in Figure 1C,D. A detailed viral infection model, which includes the immune response, was recently developed in [13]. In Figure 4 of [13], we can see that the slow decay phase II correlates to activated macrophages, i.e., the innate immune response, while the fast decay phase III correlates to a spike in CD8+ T-cells, i.e., the adaptive immune response. Using this information, we see that in a bi-phasic response, the virus is essentially controlled by the innate immune response, and the progression is mild. However, in the tri-phasic case, an adaptive immune response arises, leading to more severe cases.

Virus load functions are in high demand in the virus modeling community. For example in [14], a large community of researchers develops an individual-based SARS-CoV-2 physiological model that includes virus infection, virus transmission, immune response, and potential damage to the tissue. The immune response and the tissue complications are directly related to the virus load of the tissue. Our standard virus load function will be a welcome modeling tool to describe tissue damage and assess complication risks. Another example of detailed virus modeling is a recent study on the impact of SARS-CoV-2 on the renin-angiotensin-system by Pucci et al. [15]. A realistic virus load function is needed as model input in their model. The pharmacological company Pfizer made it their focus to develop new treatment strategies and new estimates of side effects, once a COVID-19 treatment becomes available. A virus load function, as presented here, will be a welcome tool to test their ideas. (Based on personal communication.)

## 2. Materials and Methods

### 2.1. Data Sets

We use data from five different sources: mice influenza A from [10], human rhinovirus from [16], human influenza A from [7], human SARS-CoV-2 data from [5], and Macaque monkeys SARS-CoV-2 data from [6]. Unfortunately, we do not have sex or gender information for any of these data.

In [10], 120 mice were inoculated with mouse-adapted influenza A/Puerto Rico/8/34 (H1N1) (PR8) virus, and the time series of virus load titer from the lungs were measured (10 observation per time point). For each measurement, a mouse had to be sacrificed; hence, the data are not longitudinal for individual mice. Initial conditions for the virus-target model, see below, are also available in that reference. The data are shown, together with our fits, in Section 3.

In [16], 24 patients were inoculated with 300 TCID${}_{50}$ of rhinovirus (RV-16), and nasal washes were collected to determine copies of viral RNA. The patients were grouped into three groups: a non-asthmatic control group of 8 patients, a group of 10 asthmatic patients with low Immunoglobulin E levels (IgE), and 6 asthmatic patients with high IgE levels. Data were collected eight times during the 21 days of the experiment (days 1, 2, 3, 4, 7, 10, 14, and 21) and averaged for each group. We show the reported geometric mean per group at the given time points together with our fits in Section 3.

In [7], six patients were inoculated with wild-type human influenza A/Hong Kong/123/ 77, and nasal washes were collected daily after 24 h until day eight. The original data came from an earlier study [17] and they were used before to study models for viral kinetics [18,19]. We show the individual patient data, and our fits, in Section 3.

In [5], a cohort of COVID-19 patients from two Hong Kong hospitals was evaluated. A total of 30 patients were screened between 22 January 2020 and 12 February 2020, and 23 patients were included in the study. For each patient, on a daily basis, a multitude of clinical measurements were recorded, including a virus-load measurement. For patients who were not intubated, an oropharynx saliva sample was collected. In the early mornings, patients were asked to cough up to clear the throat, and the virus load in the saliva was measured. From patients who were intubated, a endotrachial aspirate sample was taken. As the ciliary activity of the lung epithelium transports mucus to the posterior oropharyngeal area, these samples give a good indication of the viral activity in the lungs. The data were collected on a daily basis and recorded as mean values and standard deviations. We show the individual patient data for eight patients, and our fits, in Section 3.

In [6], nine rhesus macaques monkeys were infected with SARS-CoV-2. Three monkeys (Group 1) obtained a high initial virus dose, three monkeys (Group 2) a medium initial dose, and three (Group 3) a small initial dose. The virus load was measured daily or every other day through a bronchoalveolar lavage probe. All nine monkeys showed only mild disease symptoms and they all fully recovered. Hence the infection cycle here is more indicative of a mild infection, in contrast to the human data considered above. We show the individual data for the nine monkeys, and our fits, in Section 3.

### 2.2. Data Fitting Procedure

We fit the virus load function (1) to the five data sets presented above. The virus load titer is measured as a relative RNA expression, as compared to a reference gene. Hence, the measurements have a significant measurement threshold ϑ, and virus loads below this threshold cannot be seen. This threshold is 0 (on a logarithmic scale) for the mice influenza A data, assumed to be 0 for the rhinovirus data, 0.5 in the human influenza A data, 1 for the human SARS-CoV-2 data, and 1.7 for the macaque monkey SARS-CoV-2 data. Hence, measurement values at the threshold cannot be used for the fitting of the curves since the virus load might be lower than recorded. To account for this, we fit the data to the *effective virus load function* as follows:

(5) $$V_{\vartheta }\left(t\right)=\max\left\{V\left(t\right),\vartheta \right\},$$

where ϑ is the detection threshold given by the data. This explains why the fitted curves in Figures 7 and 8 seem to “ignore” the non-detection values that are shown for large times. We also assume that each subject starts and ends with a viral load equal to the threshold. Hence, we force those values if necessary. We also ignore subjects with fewer than 5 observations.

To fit the data, we use the Levenberg–Marquard algorithm available in the *LsqFit.jl* package for Julia. The initial guess for $a_{1}$, $a_{2}$, $b_{1}$, and $b_{2}$ is taken from an ordered sample of four values uniformly distributed between the 0 and the maximum time of the experiment. The lower and upper bounds are set to be evenly spaced around those initial guesses. The initial guess for $V_{\max}$ is taken as the maximum value in the data $\times 10^{\pm 3}$ to define the bounds. The value of $V_{\min}=10^{-7}$ is fixed. Finally, the initial guess for α is a random number between 0 and 1 with α bounded between $\left[10^{-8},10^{4}\right]$. We found the global minimum by starting from ten thousand random initial guesses and choosing the fit with lowest residual sum of squares.

When fitting the viral load function to each subject separately, we compute the set of parameter values that provide a similar residual sum of squares (RSS), compared to the best fit within relative tolerance ϵ:

(6) $${\hat{\Theta }}_{\epsilon }=\left\{\theta ={\left(a_{1},a_{2},b_{1},b_{2},\alpha ,V_{\max}\right)}^{t},\;\left|\;\frac{\mathrm{RSS}\left(\theta \right)-\mathrm{RSS}\left(\hat{\theta }\right)}{\mathrm{RSS}(\hat{\theta })}\right|\leq \epsilon \right\},$$

where

$$\mathrm{RSS}\left(\theta \right)=\sum_{i}{\left(\log V_{\vartheta }\left(t_{i};\theta \right)-\log v_{i}\right)}^{2},$$

and $\hat{\theta }$ is the best parameter estimate that minimizes the residual sum of squares. Relative tolerance ϵ is equivalent to $\mathrm{RSS}\left(\theta \right)\leq \left(1+\epsilon \right)\mathrm{RSS}\left(\hat{\theta }\right)$. We choose $\epsilon =-\frac{2}{n}\ln\left(0.15\right)$, where *n* is the number of data points. This is equivalent to the likelihood intervals if we assume normally distributed errors with mean zero and variance $\sigma^{2}$, and we use the maximum likelihood estimator ${\hat{\sigma }}^{2}=RSS\left(\hat{\theta }\right)/n$ [20,21]. A 0.15 likelihood interval can be interpreted as a 95% confidence interval [20]. To compute the likelihood region $\hat{\Theta }$, we sample $10^{6}$ parameter values from a normal distributions with the mean equal to the best estimate $\theta_{i}$ and standard deviation $s_{i}$, i.e.,

$$\theta_{i}∼N\left({\hat{\theta }}_{i},s_{i}\right),\;i=1,\cdots ,6,$$

and keep suitable parameters in ${\hat{\Theta }}_{\epsilon }$. We choose $s_{i}=1.1$ for $a_{1}$, $a_{2}$, $b_{1}$, and $b_{2}$, and $s_{i}=0.5$ for α and $\log V_{\max}$. The range of possible virus load curves that arises from choosing parameters in the likelihood range ${\hat{\Theta }}_{\epsilon }$ is indicated as a red cloud in Figures 5–8 below.

### 2.3. Hyperbolic Tangent

The hyperbolic tangent function,

$$\tanh\left(x\right)=\frac{e^{x}-e^{-x}}{e^{x}+e^{-x}},$$

is a sigmoid step function that smoothly transitions from −1 to 1. In (1), it is shifted and scaled such that transitions occur between $a_{1}$ and $a_{2}$ upwards and between $b_{1}$ and $b_{2}$ downwards, where the maximum is $V_{max}$ and the minimum is $V_{min}$.

**Figure 2:**
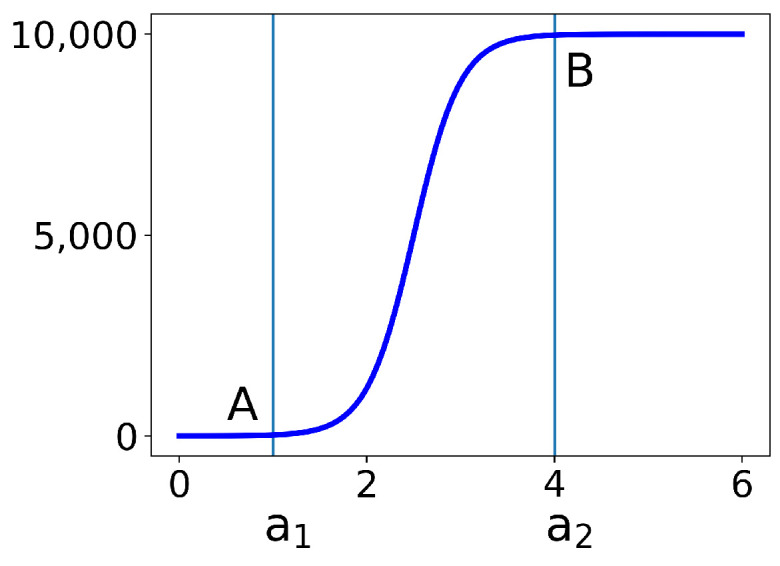
The first part $v_{1}\left(t\right)$ of the virus load function (2) to show the qualitative features, while the function transitions from 0 to $V_{max}$ between $a_{1}=1$ and $a_{2}=4$. At points A and B, it assumes 99.5% of the limit values on the left or the right, respectively.

In Figure 2, we plot the first part $v_{1}\left(t\right)$ from (2) for the choices of $a_{1}=1,a_{2}=4$ and $V_{max}$ = 10,000. At point *B*, the function has reached 99.5% of its saturation value. Indeed, if we set $x=a_{2}$, then we have the following:

$$\tanh\left(\frac{6}{a_{2}-a_{1}}\left(a_{2}-\frac{a_{1}+a_{2}}{2}\right)\right)=\tanh\left(3\right)=0.995.$$

Similarly, at point A at $a_{1}$, the function is 0.5% above its minimum.

### 2.4. Computation of Exponential Growth Rates

In [22], it was explained that it is important to estimate the exponential growth rate during the initial growth phase (Phase I), as well as the exponential decay phase in Phase II. The exponential decay rate during Phase II is a direct model parameter in our virus load function −α, which we estimate for each patient. To estimate the growth rate of the initial phase, we consider $v_{1}\left(t\right)$ from (2). We ignore the constant offset 1, and compute the logarithmic derivative of $v_{1}\left(t\right)-1$ as

$$\frac{d}{dt}\log_{10}\left(v_{1}\left(t\right)-1\right)=\frac{6}{\ln\left(10\right)\left(a_{2}-a_{1}\right)}\;\frac{{\mathrm{sech}}^{2}\left(\frac{6}{a_{2}-a_{1}}\left(t-\frac{a_{1}+a_{2}}{2}\right)\right)}{\tanh\left(\frac{6}{a_{2}-a_{1}}\left(t-\frac{a_{1}+a_{2}}{2}\right)\right)+\tanh\left(3\frac{a_{1}+a_{2}}{a_{2}-a_{1}}\right)}.$$

**Figure 3:**
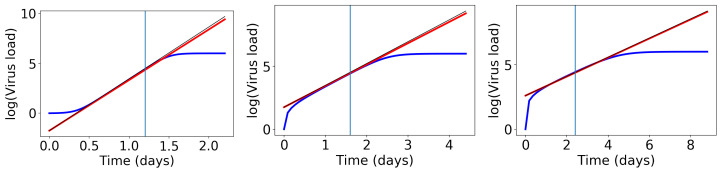
Comparison of exponential growth rates at the initial growth phase. The blue line shows $\log(v_{1}\left(x\right))$, the red line a linear approximation with slope (7), and the thin black line a linear approximation with slope (8). Left: $a_{1}=1,a_{2}=2$, middle: $a_{1}=1,a_{2}=4$, right: $a_{1}=1,a_{2}=8$.

We conducted a number of numerical tests (examples in Figure 3) and we found that the slope of the exponential growth phase is best approximated if we evaluate the above derivative at the weighted average $\bar{t}=0.8a_{1}+0.2a_{2}$. Then, the exponential growth rate becomes the following:

(7) $$\begin{array}{ccc} \lambda & = & \frac{d}{dt}\log_{10}\left(v_{1}\left(t\right)-1\right){|}_{\bar{t}}\\ & = & \frac{6}{\ln\left(10\right)\left(a_{2}-a_{1}\right)}\;\frac{{\mathrm{sech}}^{2}\left(-3.6\right)}{\tanh\left(-3.6\right)+\tanh\left(3\frac{a_{1}+a_{2}}{a_{2}-a_{1}}\right)}. \end{array}$$

The straight lines with this slope are shown as red lines in Figure 3. This formula works fine, but it is too complicated to learn anything from it. Hence, we perform an approximation. Remember that earlier, we showed that tanh(3)=0.995, which is close to 1. Since $\left(a_{2}+a_{2}\right)/\left(a_{2}-a_{1}\right)>1$, the value $\tanh(3\frac{a_{2}+a_{1}}{a_{2}-a_{1}})$ is even closer to 1. Hence, we approximate as the following:

(8) $$\lambda \approx \frac{6}{\ln\left(10\right)\left(a_{2}-a_{1}\right)}\;\frac{{\mathrm{sech}}^{2}\left(-3.6\right)}{\tanh(-3.6)+1}\approx \frac{5.2}{a_{2}-a_{1}}.$$

This approximate slope is shown as a thin black line in Figure 3.

This last formula (8) is a convenient way to estimate the exponential growth rate of Phase I. We just need the two time points of viral onset $a_{1}$ and viral saturation $a_{2}$, and we obtain an estimate for λ, which is as good as a full fit with the ODE model (see Figure 1C,D). We can use the same formula with $a_{1},a_{2}$ replaced by $b_{1},b_{2}$ to estimate the decay rate in Phase III.

### 2.5. Viral Target Model

There is extensive mechanistic modeling of viral load curves based on ordinary differential equation models [7,8,18,23,24]. For example, Baccam et al. [7] developed a four-compartment virus-target model to describe the virus load in a given person. This target model has been extended in many different directions, including eclipse and saturation terms [8,18,25,26,27,28,29,30], different anti-viral treatments [29,31,32,33], competing virus infections [25,34], immune responses [26,28,35], and correlations of viral load with disease severity [4,36]. Most of this research happened during the past year, stimulated by the COVID-19 pandemic. In Section 3, we compare this more traditional approach to our virus load function.

The *target model* with the eclipse phase comprises four ordinary differential equations (ODEs) for the target cells T(t), the infected cells $I_{1}\left(t\right)$, the infectious cells $I_{2}\left(t\right)$, and the virus load V(t) as follows:

(9) $$\begin{array}{cc} \dot{T} & =-\beta TV\\ {\dot{I}}_{1} & =\beta TV-kI_{1}\\ {\dot{I}}_{2} & =kI_{1}-\frac{\delta_{d}I_{2}}{K_{d}+I_{2}}\\ \dot{V} & =pI_{2}-cV. \end{array}$$

Here, β is the virus infection rate, *k* the rate at which infected cells become infectious, *p* the virus production rate, *c* the virus decay rate, $\delta_{d}$ the base decay rate of infectious cells, and $K_{d}$ the half saturation constant for the decay term of the infectious cells. This model is an improvement of the standard viral kinetic model [7] in which the authors introduce a saturation term for the infected cell clearance ($\frac{\delta_{d}}{K_{d}+I_{2}}$) in the equation for $I_{2}$ to describe a tri-phasic virus growth and decay [10]. The basic reproduction number $R_{0}$ for this model is given as follows (see [10]):

(10) $$R_{0}=\frac{\beta pK_{d}T\left(0\right)}{c\delta_{d}}.$$

Typical outcomes of the target model are shown in Figure 1C,D as a blue line, and the model parameters and their meaning is summarized in Table 2. The parameters of Figure 1C,D have not been fit to any data and are simply chosen to highlight the different cases of the bi-phasic and tri-phasic response. In Section 3, we fit the target model to the influenza data.

## 3. Results

We fit our virus load function (1) to the virus load data of infection of influenza A for mice and humans, rhinovirus for humans, and in SARS-CoV-2 data for humans and Macaque monkeys. Details on these data sets and our data fitting procedure is explained in Section 2. Furthermore, we compare this new virus load function (1) to the standard target model (9) for viral kinetics [10].

### 3.1. Mice Influenza A Data

**Figure 4:**
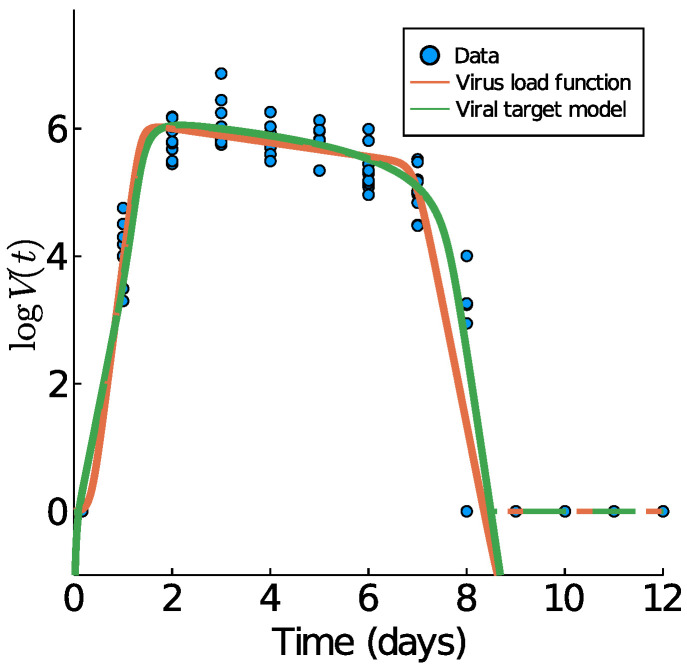
Fitting results of the virus load function (1) in orange and the virus-target model (9) in green to influenza A data of mice [10]. The effective virus load curve (5) for both models are indicated with dashed lines.

We begin with Influenza A virus load data from [10], as these are the best experimental data available, based on tightly controlled murine experiments. In Figure 4, we show those data plus the data fitting results of the virus load function (1) and the viral target model (9). The fit of the target model to these data was previously performed by Smith et al. in [10].

The corresponding model parameters of the virus load function (1) are listed in Table 2. Of particular importance is the virus-load decay rate in Phase II. Our virus load function estimates the negative slope as $-\alpha =-0.26\;d^{-1}$, quite similar to the estimate in [10], using linear regression for the middle portion ($-0.2\;d^{-1}$). The virus load function estimates the duration of Phase I at approximately 2.41 days, Phase II at 3.22 days, and Phase III at 1.35 days. The residual sum of squares for the virus load function and the viral target model are 36.1923 and 35.7378, respectively.

The estimated slopes are λ=9.08 $\log_{10}$TCID${}_{50}$/d in Phase I, −α=−0.26 $\log_{10}$TCID${}_{50}$/d in Phase I, and −3.77 $\log_{10}$TCID${}_{50}$/d (using (8) for Phase III) in Phase III. Our estimates in the decays phases are the same to those reported in [10] (−0.2 $\log_{10}$TCID${}_{50}$/d in Phase II and −3.8 $\log_{10}$TCID${}_{50}$/d in Phase III) are overestimated for the growth Phase I (4.7 $\log_{10}$TCID${}_{50}$/d). This overestimation occurs because of the tendency of the virus load function to have a horizontal slope at t=0.

### 3.2. Human Rhinovirus Data

**Figure 5:**
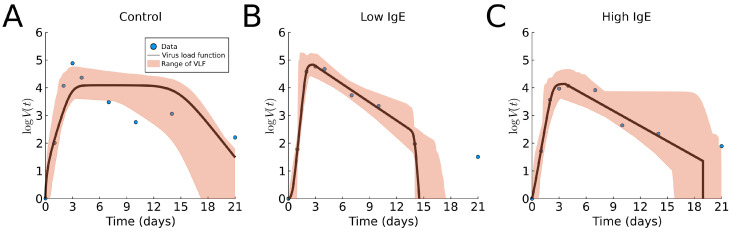
Fitting results of the virus load function (1) to Rhinovirus data [16]. (**A**) shows the non-asthmatic control group, (**B**) the asthmatic group with low IgE, and (**C**) the asthmatic group with high IgE. The blue dots represent the group average data; the black line is our best fit. The area in red represent the likelihood range of the virus load functions.

Now, we fit the virus load function (1) to the three groups of human rhinovirus data [16]. The results are shown in Figure 5 and Table A1. We observe that the virus load function did not capture a slow decay phase in the control case (α=0), perhaps due to fact that the data show that the last portion (days 6 to 21) has a slower decay rate than that of the intermediate portion (days 3 to 6). We find slightly lower maximum viral load and longer infections in the asthmatic group with high levels of total IgE, compared to the asthmatic group with low levels of total IgE. These findings are similar to those using cumulative viral load (data not available) [16]. We reiterate that it is not clear from the data that the decay in the viral load is tri-phasic with a slow decay followed by a faster decay; instead, it appears that the tri-phasic decay is reversed, with a slow decay followed by a slower decay.

### 3.3. Human Influenza A Data

**Figure 6:**
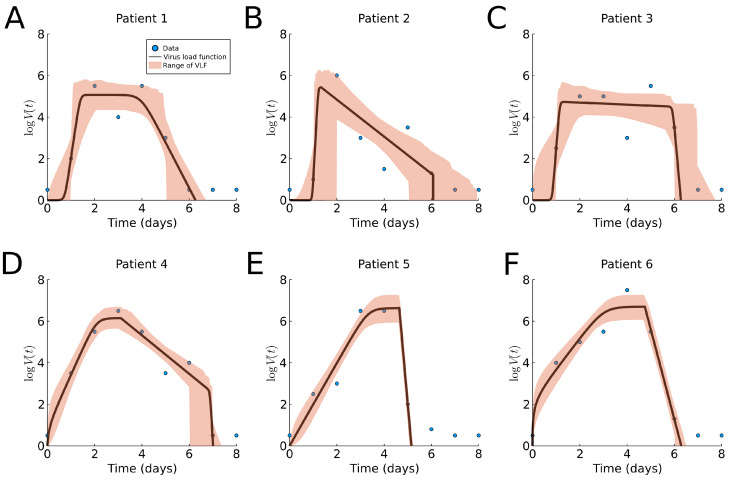
Fitting results of the virus load function (1) to human Influenza A data data [7]. The blue dots represent the individual measurements, and the black line is our best fit. The area in red represents the likelihood range of virus load functions. (**A**–**F**) Patients 1 to 6, respectively.

In both previous cases, the virus load function was fitted to average data. We now fit data to individual subjects to show the benefits of patient specific fitting. The results of fitting the virus load function to human influenza A data are shown in Figure 6 and Table A1, and a boxplot for the estimated parameter values is shown in Section 3.6. We observe noticeably different profiles among patients, with the exception of Patients 5 and 6. Although estimation of the onset of the growth phase is similar in all six cases ($a_{1}\approx 1$), the time of saturation, slow decay rate and time of the third phase vary across patients. The cloud of likely virus load functions shows three type of responses, (1) slow decay rate in Phase II (Figure 6A,C), (2) faster decay rate in Phase II (Figure 6B,D), and (3) very fast decay in Phase II, leading to clearance and absence of Phase III. This indicates that patients 5 and 6 show a bi-phasic profile (monophasic decay).

We compare the approximate slope in Phase I, λ from (8), with the approximate growth exponent as reported in [22]. We report them as “value from Table A1 (value from [22])”. For Patients 1 and 2, we find a good match 7.69(8.76) and 20.93(18.83). For Patient 3, we find a larger slope 14.44(6.94); however, the lower slope of 6.94 is well within our error tolerance. For Patients 4, 5, and 6, 2.53(6.46),1.98(5.08), and 1.49(6.20), our estimate is systematically lower. This is related to the fact that our approach finds a later viral load maximum as compared to the ODE model in those cases.

### 3.4. Human SARS-CoV-2 Data

**Figure 7:**
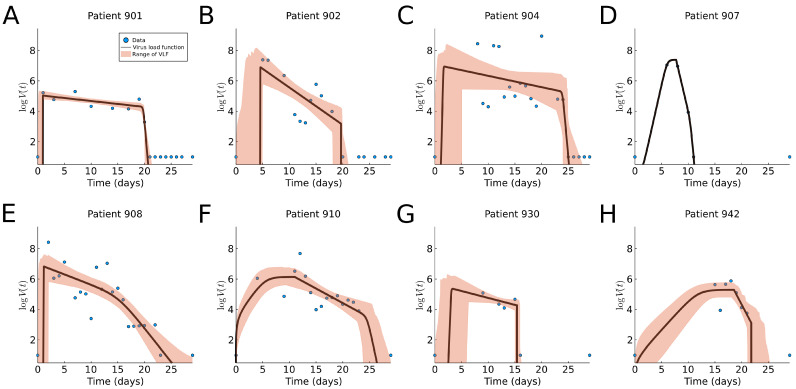
Fitting results of the virus load function (1) to human SARS-CoV-2 data [5]. The blue dots represent the individual measurements and the black line is our best fit. The area in red represents the likelihood range of virus load functions. (**A**–**H**) Fitting to patients 901, 902, 904, 907, 908, 910, 930, and 942, respectively. The black curve is the nonlinear square fit, and the shaded region indicates virus load curves with RSS within relative tolerance ϵ=0.2.

We fit our virus load function (1) to eight patients in this data set as shown in Figure 7. The corresponding model parameters and their ranges are listed in Table A1, and a boxplot for the range of parameter values is shown in Figure 9b. We observe that the virus load over time varies greatly across patients; some show long infection periods (20–25 days), while others are short (∼10 days). The virus load function is able to describe the three phases of the virus for most of the patients (901, 902, 904, 908, and 930). In those patients, we observe that the initial virus growth phase is rather quick, and the virus reaches its carrying capacity within a day ($a_{2}-a_{1}<0.87$), which means that the slope Phase I is large (λ>5.97). The slope during the second phase varies from −0.9 to −0.57. The virus load reaches a saturation level, which is likely to be related to the innate immune response, and it starts a phase of slow decay with a half-life time between $T_{1/2}=1.22$ and 5.33 days. After 15 days, the virus load drops more quickly, possibly due to the adaptive immune response, and at day 25, the virus is cleared. Note that this is sensitive to missing data. For instance, in patients 902, 904, 907, 930, and 942, there are no data at the beginning for several days. Note also that, for example, for patient 930, there is a sudden full clearance of the virus at day 15. As consequence, $b_{1}$ and $b_{2}$ are estimated to be equal.

The shaded areas in the plots of Figure 7 are able to pick up areas of missing values that affect the estimated times of the corresponding phases. For example, missing information of the time of infection for patients 901, 902, 904, 908, and 930 results in a wider cloud at the initial times.

For patient 907, the fit is exact, but it should be noted that in this case, we have more parameters than data points, and a good fit is not very meaningful. For Patient 930, we have very few data points, but they seem to be more scattered to obtain an exact fit. Patients 904, 930 and 942 show an extended initial Phase I, which spans over 8 days for Patients 904 and 930 and 10 days for Patient 942. This is, of course, related to the missing initial information about the early infection times.

We see that the virus load function (1) describes all virus load curves very well. It is easily adapted for long and short virus infection periods, and it is fully flexible in detecting all three phases of the viral progression. Using the tolerance cloud, we can also visualize regions of greater uncertainty due to missing data points. We will see that the dynamics for macaque monkeys is rather similar.

### 3.5. Macaque Monkey Data

**Figure 8:**
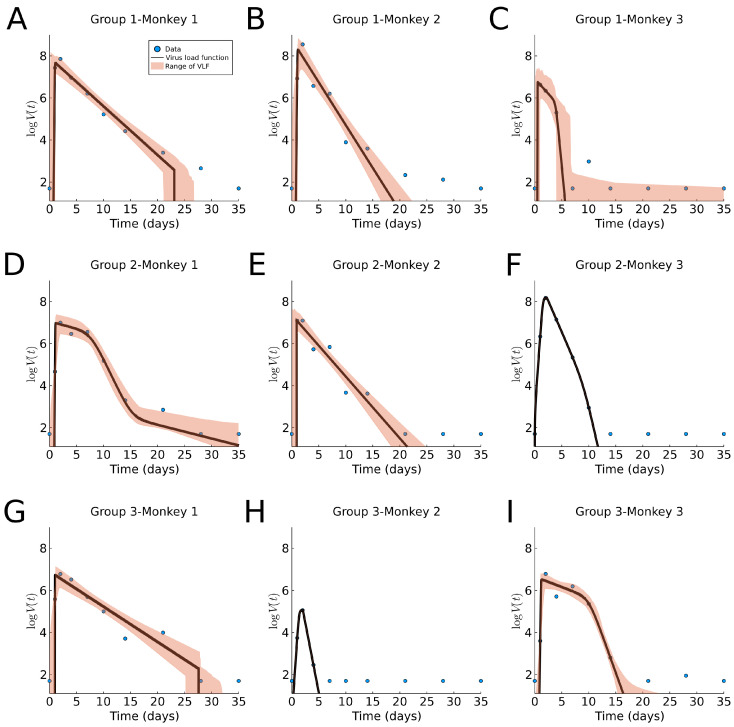
Fitting results of the virus load function (1) to macaque monkey SARS-CoV-2 data [6]. The area in red represent the likelihood range of virus load functions. (**A**–**I**) Groups 1, 2, and 3, Monkeys 1, 2, and 3, respectively.

**Figure 9:**
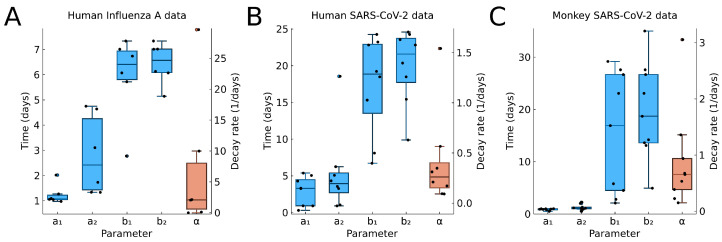
Boxplots for the estimated parameters using (**A**) human influenza A data, (**B**) human SARS-CoV-2 data from oropharynx saliva samples, and (**C**) macaque monkey SARS-CoV-2 data. Time-related parameters are shown in the blue boxplot, and the decay rate is shown in the red boxplot with a separate axis on the right. Points indicate the individual best estimate shown in Figure 6, Figure 7 and Figure 8, and in Table A1.

In Figure 8, we fit our virus load function (1) to the rhesus monkey data, and we report the parameter values and ranges in Table A1 and a boxplot in Figure 9C. The characteristic values for the initial virus growth $a_{2}<2.3$ is common between all monkeys, indicating that the amount of the initial viral dose is not so important. The virus load is bi-phasic in most monkeys (1-1, 1-2, 2-2, 2-3, 3-1, and 3-2) in which the rate of decay is larger (>0.6 days${}^{-1}$, and half-life time <1.2 days). For the remaining monkeys (1-3, 2-1, and 3-3) a fast decay phase is observed, following a slow decay with a smaller decay rate. This occurrence of the bi-phasic viral load dynamics suggests that in some monkeys, the action of the immune system is rather efficient. Additionally, note that the estimated range for the parameters is small for $a_{1}$, $a_{2}$ and α and larger for $b_{1}$, $b_{2}$, $V_{\max}$ (see also Figure 9C). Interestingly, the fit for monkey 1 in Group 2 shows an extra last phase with a slow exponential decay. This occurs when the slope α is small and the times $b_{1}$ and $b_{2}$ are far apart, causing the slope in this extra phase to be approximately alpha.

Compared to the human data, we notice that the virus half-life times in Phase II is $T_{\frac{1}{2}}>1.2$ days in both human and monkeys experiencing all three phases, and $T_{\frac{1}{2}}<1.2$ days in those that show a bi-phasic behavior. Hence, a bi-phasic behavior is indicative of faster virus clearance in Phase II. The length of the infection is estimated as about 27.5 days for humans and 34 days for the monkeys, where the final decay phase starts significantly earlier in humans $b_{1}=15$ days than in monkeys $b_{1}$, ranging 25–30 days. This could be an indication of a more efficient adaptive immune response in humans as compared to monkeys (see also [13]).

### 3.6. Parameter Distributions

In Figure 9, we show the boxplots for the time values $a_{1}$, $a_{2}$, $b_{1}$, $b_{2}$, (blue) and the decay rate α (red) from the fitting results to individual data. For $a_{1}$, we see very little variability in A and C. These correspond to controlled experiments, where the time of initial infection is known. If the time of initial infection is not known, such as in B, then we have greater variation in $a_{1}$.

Overall, we see a large variability of the model parameters, in particular, the values $b_{1},b_{2}$, which indicate the end of the infection.

For the decay rate α (red in Figure 9), we see also great variability. The values for human and macaque viral infection are comparable, indicating that macaque are a useful model species.

### 3.7. Comparison to the Virus-Target Model of Smith et al.

As mentioned in the introduction, the virus load modeling with ordinary differential equations is a well-developed field [8,18,23]. Here, we like to compare our virus load function (1) with one of the most current target models: model (9) of A. Smith et al. [10].

In Figure 1C,D, we show the virus load curve from the target model as a blue line, showing a tri-phasic and bi-phasic type, respectively, in virus growth and decay, using parameters values listed in Table 2. Note that the corresponding $R_{0}$ values (10) are significantly different and higher for the tri-phasic case. To these curves, we fit the virus load function (1) using data points every 0.1 days. The fits are show in Figure 1 as red curves. We see that the virus load function (1) can reproduce both bi-phasic and tri-phasic curves with a high level of accuracy. The best estimate for the parameter values are listed in Table 1 (except for $V_{\min}$, which is a fixed value).

Fitting the virus-target model (9) to data constitutes statistical and numerical challenges. For instance, initial conditions are unknown in most cases, and estimating these values has shown to be impractical [10]. However, it is easier to fit the virus load function (1) to virus load data due to the empirical nature of the parameter in the function.

## 4. Discussion

The explicit form of this new standard virus load function (1) is simple and convenient.

The purpose of the function is to model a viral infection in an individual showing a fast exponential increase in the virus load, followed by an initial slow and later fast exponential decrease. Our virus load function is not intended to replace the Baccam or target model approach. Indeed, the target model has been used with great success in many applications and experiments [4,7,8,18,23,24,25,26,27,28,29,30,31,32,33,34,35,36]. Rather, the virus load function is an alternative description. Instead of using rates of growth, infection, and clearance, we use time points, such as the point of onset of viral growth $a_{1}$, time to maximum $a_{2}$, the beginning of the fast decay phase $b_{1}$ and the time of virus clearance $b_{2}$. As such, these values are very intuitive, as they have easily understandable biological meaning, allowing this model to be used quickly and efficiently.

We have shown that this virus load function can replicate observed virus load titers from Influenza A in mice [10] and humans [7], rhinovirus in humans [16], and from SARS-CoV-2 in humans [5] and monkeys [6]. The virus load function gives direct information about the time course of the viral infection periods. In addition, it is very efficient at estimating the exponential growth and decay rates of the various Phases I, II, and III. To estimate the initial growth rate, we use the simple formula (8), and we have shown above that this growth rate fits the data very well (see the dashed lines in Figure 1C,D). The decay rate for Phase II −α is a direct model parameter; hence, it describes the intermediate decay accurately (see Figure 1C,D). Finally, for Phase III, we use formula (8) with $a_{1},a_{2}$ replaced by $b_{1}$ and $b_{2}$ to estimate the fast decay rate (again, see Figure 1C,D).

The virus load function is able to fit data, having either a bi-phasic or tri-phasic profile, as seen in the human influenza A and monkey SARS-CoV-2 data. The bi-phasic infection is characterized by a fast decay in the viral load after reaching the peak. The tri-phasic profile is characterized by a slow decay in the viral load followed by a fast clearance of the virus. We believe that the distinction of the bi-phasic and tri-phasic is a first indication of disease severity. In [37], a correlation was made between disease severity and the area under the virus load curve (AUC). The AUC is a measure for the total virus attack on the host. As the tri-phasic viral load response has a larger AUC than a corresponding bi-phasic curve, a tri-phasic response is indicative of a more severe outcome. Smith [10] correlates severity to the slope α of Phase II. A slower decaying viral load leads to a more severe outcome, again relating severity to a tri-phasic response. This effect is further explained in [38], where a slow intermediate virus decline indicates a complex cytokine-immune response activation that includes broad-band innate immune response, more severe tissue damage, increased thrombosis and cytokine storms. In [13], a measure for viral severity in the form of an inflammation function is introduced. This inflammation function quantifies severity based on the processes of viral infection and immune responses. In future studies, we will explore this idea further to formally connect bi- and tri-phasic behaviors with disease severity.

Our model fitting to data shows a large patient-to-patient variability, as expected (see Table A1). The likelihood range of virus load functions behaves well in the case of missing data points. For example, Patients 902, 904, 910, 930 and 942 in Figure 7 did not have an initial measurement before the viral load maximum. In this case, we see an enlarged cloud of uncertainty near the viral onset.

The approximate slope of Phase I, λ, shown in Table A1 has large variability in general. This is due to the small difference in the estimated values of $a_{2}$ and $a_{1}$ in some individuals. They correspond to individuals with large overlapping likelihood intervals. For example, in Patient 2 in the human influenza A data, Patient 901 in the human SARS-CoV-2 data, and Monkey 2 in Group 2 in the monkey SARS-CoV-2 data, the likelihood intervals for $a_{1}$ and $a_{2}$ overlap in more than 50% of the interval. This shows that one of the issues with the virus load function is the uncertainty in the estimations of $a_{1}$ and $a_{2}$ when there is no observation to characterize Phase I. The same problem occurs in Phase III.

We understand the large patient-to-patient variability as confirmation that patient-specific modeling is useful. The large variation in the values for $b_{1},b_{2}$ for humans and macaque monkeys (see Table A1 and Figure 9), for example, indicates that many different processes are at play in a complicated interaction, such as immune responses, metabolism, and cytokine signaling [13]. The clear distinction of the three phases coincides well with the expected onset of the innate immune response near time $a_{2}$ and the adaptive immune response near time $b_{1}$ [10,13]. However, more research is needed to establish a clear correlation.

The virus load function is specifically designed for acute virus infections, such as influenza and corona viruses. It is not expected to be useful for viral infections with a different profile, such as HIV, for example [39].

A few notes are worth considering when fitting the virus load function to individual data. Firstly, the measurement threshold for viral titer needs to be taken into account, e.g., by fitting to the effective virus load function (5). Secondly, an initial parameter estimate can be gained by a simple visual inspection of the viral load data. Then, the parameter space should be tested to guarantee a global minimum. Thirdly, the range of virus load functions obtained forms some parameter interval estimations (some as likelihood or confidence intervals), which should be computed to observe a family of possible virus load curves that reflect the uncertainty on the data. We find the 0.15 likelihood intervals (6) by computing the residual sum of squares within relative tolerance [20]. Alternatively, credible intervals can be obtained using a Bayesian approach, but we leave this task as future work [40].

The virus load function (1) can be used in future work in many different ways. In [37], the target model is considered in the context of various viral therapies and we can perform a similar discussion here. A reduction in the viral infectivity, for example, through amantadines [37], will result in a shorter infection period (reduced $a_{2}$), which consequently reduces the viral load maximum and expedites virus clearance. A reduction in viral reproduction inside cells, for example, through neuraminidase inhibitors [37], will also shorten the initial growth phase, i.e., reduce $a_{2}$. An increase in the viral clearance rate, for example, through monoclonal antibodies [37], will take the most effect at Phases II and III, where the virus is cleared. The value for α would be increased (faster decay), and the time point $b_{2}$ would arise earlier. Immune therapies would be expected to have two effects. The immune response might be faster than normal i.e., $a_{2}$ is reduced, and the viral clearance might be faster, i.e., α is increased. Finally, vaccination is a pre-conditioning of the immune response, which can act quickly once a real infection occurs. In this case $a_{2}$ will be reduced drastically, eliminating the virus, even before it can establish itself.

If models for the immune response are considered explicitly, then the virus load function can be used as a model input, allowing us to correlate the time parameters $a_{1},a_{2},b_{1},b_{2}$ with typical immune response times. Acute viral infections, such as SARS, SARS-CoV-2, MERS and others, are known to affect the body system widely [1,2]. Not only the lung tissue is infected, but secondary complications arise in the heart, the circulatory system, the kidneys, the digestive system and the brain [1,2]. The secondary effects considerably increase the severity of the disease [1,2]. We plan, in future work, to use the virus load function as input into tissue damage models for the heart, blood circulation, the brain and others, and to establish a risk index for individual patients.

## Figures and Tables

**Table 1:** Parameters of the standard virus load function (1) corresponding to Figure 1A–D. The virus load curves reported in [10] are used in (a) and (b).

Parameter	A, B	C	D	Meaning (Units)
$V_{\max}$	$10^{6}$	$9.4\cdot 10^{5}$	$1.6\cdot 10^{5}$	maximum virus load (TCID${}_{50}$)
$V_{\min}$	$10^{-7}$	$6\cdot 10^{-8}$	$5.2\cdot 10^{-3}$	minimum virus load (TCID${}_{50}$)
$a_{1}$	0.5	0.9	2.2	onset of virus growth (d)
$a_{2}$	4	2.07	5.1	enter virus saturation (d)
α	0.1	0.38	1.3	intermediate decay rate (d${}^{-1}$)
$b_{1}$	13	6.9	16	onset of rapid decay (d)
$b_{2}$	19	7.9	23.8	reach virus clearance (d)

**Table 2:** Parameters of the target model (9) corresponding to data Figure 1C,D and data fit in Figure 4. Fixed parameters are denoted with *.

Parameter	C	D	Figure 4	Meaning (Units)
β	$9.9\cdot 10^{-5}$	$9.9\cdot 10^{-5}$	$2.78\cdot 10^{-5}$	virus infection rate (TCID${}_{50}^{-1}$ d${}^{-1}$)
*p*	1.7	0.8	1.66	virus production rate (TCID${}_{50}$ cell${}^{-1}$ d${}^{-1}$)
*c*	12.48	12.48	13.58	virus decay rate (d${}^{-1}$)
*k*	4	4	4 *	infection maturation rate (d${}^{-1}$)
$\delta_{d}$	$1.65\cdot 10^{6}$	$1.05\cdot 10^{7}$	$1.53\cdot 10^{6}$	base decay rate of infect. cells (cell${}^{-1}$ d${}^{-1}$)
$K_{d}$	113,400	113,400	31,280	half saturation constant (cells)
T(0)	$10^{7}$	$10^{7}$	$10^{7}$	*T* initial condition (cells)
$I_{1}\left(0\right)$	75	75	75	$I_{1}$ initial condition (cells)
$I_{2}\left(0\right)$	0	0	0	$I_{2}$ initial condition (cells)
V(0)	0	0	0	*V* initial condition (cells)
$R_{0}$	9.268	1.134	6.948	basic reproduction number

## Data Availability

The data used in this research were kindly provided by the authors of [5,6,10].

## Appendix A

Tables with the data fitting results are shown here.

**Table A1:** Estimated parameter values corresponding to data fitting in Figure 5, Figure 6, Figure 7 and Figure 8. For each subject, the first row is the best estimate, and the second row corresponds to the likelihood intervals. The RSS column shows the minimum RSS value and the maximum tolerance, respectively. The λ column shows the approximate slope of Phase I, according to (8).

Data	$\log V_{\max}$	$a_{1}$	$a_{2}$	α	$b_{1}$	$b_{2}$	RSS	λ
Group	Mice influenza A							
Mice	6.05	0.94	1.52	0.26	6.25	7.63	36.192	9.08
	(5.92, 6.11)	(0.91, 0.99)	(1.15, 1.97)	(0.19, 0.34)	(6.16, 6.33)	(7.57, 7.73)	≤37.282	–
Group	Human rhinovirus							
Control	4.10	0.34	5.30	0.00	8.33	21.00	5.306	1.05
	(3.48, 4.89)	(0.00, 1.36)	(1.47, 6.72)	(0.00, 0.56)	(3.93, 12.38)	(17.77, 21.00)	≤9.332	–
Low IgE	4.83	1.14	2.79	0.50	13.33	14.58	2.335	3.16
	(4.43, 5.31)	(0.87, 1.37)	(1.04, 3.65)	(0.25, 0.74)	(9.69, 17.51)	(14.02, 18.78)	≤4.106	–
High IgE	4.14	0.89	3.67	0.42	18.94	18.94	3.892	1.87
	(3.37, 4.69)	(0.00, 1.37)	(1.02, 6.66)	(0.00, 0.78)	(14.64, 21.00)	(16.04, 21.00)	≤6.846	–
Patient	Human influenza A							
Patient 1	5.07	1.05	1.73	0.00	2.77	5.14	1.986	7.69
	(4.36, 5.98)	(0.68, 1.39)	(1.01, 3.53)	(0.00, 1.57)	(2.13, 5.18)	(4.84, 6.23)	≤3.493	–
Patient 2	5.43	1.09	1.34	2.05	6.07	6.07	6.451	20.93
	(3.71, 6.83)	(0.81, 2.00)	(1.00, 2.74)	(0.69, 3.53)	(2.17, 8.00)	(5.02, 8.00)	≤11.346	–
Patient 3	4.73	0.97	1.33	0.12	5.72	6.13	3.675	14.44
	(4.07, 5.92)	(0.00, 1.28)	(1.01, 4.91)	(0.00, 1.53)	(3.47, 6.99)	(6.01, 7.46)	≤6.464	–
Patient 4	6.15	1.05	3.11	2.13	6.74	7.02	1.304	2.53
	(5.63, 6.69)	(0.58, 1.36)	(2.60, 3.73)	(1.36, 3.11)	(4.78, 6.99)	(6.03, 7.81)	≤2.293	–
Patient 5	6.63	2.02	4.64	29.63	7.34	7.34	1.700	1.98
	(5.95, 7.30)	(1.57, 2.35)	(4.55, 4.73)	(27.95, 31.13)	(4.63, 8.00)	(5.25, 8.00)	≤2.990	–
Patient 6	6.70	1.27	4.75	9.98	7.01	7.01	1.606	1.49
	(6.07, 7.33)	(0.39, 1.83)	(4.49, 4.97)	(8.19, 11.90)	(4.55, 8.00)	(5.44, 8.00)	≤2.826	–
Patient	Human SARS-CoV-2							
Patient 901	5.04	0.92	0.92	0.09	19.23	20.36	0.832	Inf
	(4.84, 5.33)	(0.00, 0.99)	(0.06, 1.66)	(0.05, 0.15)	(18.07, 19.96)	(20.01, 20.86)	≤1.075	–
Patient 902	7.21	3.27	3.27	0.57	18.49	18.49	12.274	4849.14
	(5.80, 8.70)	(0.00, 5.00)	(0.55, 7.23)	(0.28, 0.85)	(14.30, 19.99)	(18.00, 22.21)	≤15.856	–
Patient 904	6.75	4.29	4.32	0.17	23.23	24.58	36.754	143.82
	(5.38, 8.53)	(0.03, 7.97)	(1.39, 9.40)	(0.00, 0.54)	(18.67, 24.99)	(23.16, 26.35)	≤44.957	–
Patient 907	7.14	0.31	3.57	0.09	8.10	9.88	0.000	1.60
	(7.14, 7.14)	(0.31, 0.31)	(3.57, 3.57)	(0.09, 0.09)	(8.10, 8.10)	(9.88, 9.88)	≤0.000	–
Patient 908	6.84	0.92	1.04	0.31	6.72	23.56	20.774	44.01
	(5.68, 7.98)	(0.00, 1.99)	(0.01, 4.42)	(0.04, 0.55)	(2.05, 11.26)	(19.16, 28.42)	≤25.153	–
Patient 910	6.74	3.32	5.08	0.35	24.26	24.26	9.211	2.96
	(5.71, 7.92)	(0.01, 4.00)	(1.59, 9.60)	(0.13, 0.55)	(19.60, 28.68)	(23.01, 28.97)	≤11.899	–
Patient 930	5.10	5.38	6.25	0.21	15.32	15.43	0.381	5.97
	(4.62, 5.89)	(1.48, 8.87)	(3.34, 9.83)	(0.06, 0.36)	(14.50, 15.98)	(15.01, 16.00)	≤0.862	–
Patient 942	5.29	5.06	18.56	1.54	22.82	22.82	2.470	0.39
	(4.94, 5.64)	(0.10, 10.23)	(17.38, 19.69)	(0.57, 3.15)	(17.91, 27.93)	(21.02, 28.16)	≤4.344	–
Group-Monkey	Monkey SARS-CoV-2							
1-1	7.66	0.88	1.10	0.53	23.08	23.08	1.407	23.29
	(7.15, 8.22)	(0.00, 1.00)	(0.02, 2.49)	(0.43, 0.64)	(19.03, 27.43)	(21.21, 27.76)	≤2.297	–
1-2	8.30	0.94	1.18	0.94	29.18	35.00	2.249	22.48
	(7.36, 9.00)	(0.00, 1.00)	(1.01, 3.04)	(0.72, 1.16)	(24.94, 33.46)	(30.91, 35.00)	≤3.672	–
1-3	6.76	0.50	0.55	0.65	2.81	4.94	1.636	108.32
	(5.96, 7.89)	(0.00, 1.00)	(0.01, 2.71)	(0.00, 2.42)	(0.28, 6.97)	(4.00, 9.72)	≤2.670	–
2-1	6.97	0.99	1.15	0.15	2.11	13.11	0.753	31.56
	(6.47, 7.39)	(0.58, 1.07)	(1.02, 3.03)	(0.02, 0.23)	(1.15, 3.22)	(11.31, 15.29)	≤1.229	–
2-2	7.10	0.91	0.91	0.68	26.67	26.67	1.310	1813.34
	(6.60, 7.78)	(0.00, 1.00)	(0.01, 2.16)	(0.55, 0.84)	(21.77, 30.81)	(23.97, 31.34)	≤2.138	–
2-3	8.18	0.78	2.25	1.36	4.51	13.63	0.000	3.56
	(8.18, 8.18)	(0.78, 0.78)	(2.25, 2.25)	(1.36, 1.36)	(4.51, 4.51)	(13.63, 13.63)	≤ 0.000	–
3-1	6.73	1.00	1.01	0.39	27.58	27.58	1.264	333.07
	(6.14, 7.15)	(0.00, 1.00)	(1.01, 3.42)	(0.29, 0.49)	(22.80, 31.28)	(24.98, 32.70)	≤2.063	–
3-2	5.06	0.65	2.04	3.05	16.91	18.70	0.000	3.75
	(5.06, 5.06)	(0.65, 0.65)	(2.04, 2.04)	(3.05, 3.05)	(16.91, 16.91)	(18.70, 18.70)	≤0.000	–
3-3	6.52	1.02	1.30	0.23	5.79	14.20	0.527	18.22
	(6.10, 6.82)	(0.94, 1.15)	(1.07, 2.69)	(0.01, 0.44)	(2.89, 8.93)	(12.55, 16.05)	≤0.859	–
